# Patterns of richness across forest beetle communities—A methodological comparison of observed and estimated species numbers

**DOI:** 10.1002/ece3.7093

**Published:** 2020-12-20

**Authors:** Nora Haack, Annegret Grimm‐Seyfarth, Martin Schlegel, Christian Wirth, Detlef Bernhard, Ingo Brunk, Klaus Henle

**Affiliations:** ^1^ Molecular Evolution & Animal Systematics University of Leipzig Leipzig Germany; ^2^ German Centre for Integrative Biodiversity Research (iDiv) Halle‐Jena‐Leipzig Leipzig Germany; ^3^ Department of Conservation Biology Helmholtz Centre for Environmental Research (UFZ) Leipzig Germany; ^4^ Systematic Botany and Functional Biodiversity University of Leipzig Leipzig Germany; ^5^ Max‐Planck Institute for Biogeochemistry Jena Germany; ^6^ Büro für Ökologische Gutachten Dresden Germany

**Keywords:** alpha diversity, Coleoptera, community patterns, floodplain forest, number of shared species, observed species number, species richness estimators

## Abstract

1. Species richness is a frequently used measure of biodiversity. The compilation of a complete species list is an often unattainable goal. Estimators of species richness have been developed to overcome this problem. While the use of these estimators is becoming increasingly popular, working with the observed number of species is still common practice.
2. To assess whether patterns of beetle communities based on observed numbers may be compared among each other, we compared patterns from observed and estimated numbers of species for beetle communities in the canopy of the Leipzig floodplain forest. These patterns were species richness and the number of shared species among three tree species and two canopy strata.
3. We tested the applicability of the asymptotic Chao1 estimator and the estimate provided by the nonasymptotic rarefaction–extrapolation method for all tree species and both upper canopy and lower canopy. In the majority of cases, the ranking patterns of species richness for host tree species and strata were the same for the observed and estimated number of species. The ranking patterns of the number of species shared among host tree species and strata, however, were significantly different between observed and estimated values.
4. Our results indicate that the observed number of species under‐represents species richness and the number of shared species. However, ranking comparisons of published patterns based on the number of observed species may be acceptable for species richness but likely not reliable for the number of shared species. Further studies are needed to corroborate this conclusion. We encourage to use estimators and to provide open access to data to allow comparative assessments.

## Graphical abstract

The observed number of beetle species considerably under‐represents species richness and the number of shared species between strata and tree species in the Leipzig floodplain forest. Still, richness ranking patterns based on the number of observed species are largely reliable. Therefore, observed species numbers can be used for rough comparative analyses of species richness but are likely not reliable for the number of shared species.

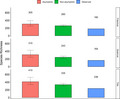

## 1 INTRODUCTION

Species richness is one of the most important measures of biodiversity. Investigations in forest canopies pose particular challenges (Barker & Pinard, 2011) that make obtaining reliable measures of species richness more complicated than in other ecosystems. This has piqued the interest of scientists for decades. Since Erwin's study on global species richness based on the data he acquired on beetles in tropical forest canopies (1982), beetles have been used as study organisms when it comes to studying species richness. Species richness is often represented as the observed number of species, even though species abundance relations are mostly skewed toward few species with many individuals and many species represented by increasingly fewer individuals (Follner & Henle, 2001). Therefore, it is usually impossible to count the actual number of species in an area. Thus, the number of observed species under‐represents the actual number of species.

Comparative analyses of communities also play an important role in ecology and for decision makers in conservation biology. A number of similarity measures exist based on the proportion of species shared between two communities (Gower, 1985; Koleff et al., 2003). In many cases, these measures are calculated using observed data. When using observed data, comparisons among sites require the assumption that the distribution of detection probabilities is the same among compared sites, which is not the case for most organisms (Chao et al., 2016b; Follner & Henle, 2001). To avoid the problems that might arise with using observed data, Chao et al. (2000), and Chao et al. (2016b) developed several estimators to improve estimates of species richness and numbers of shared species among communities.

Species richness estimators have already been used to analyze biodiversity patterns in arthropods in European forest ecosystems (Peretti & Bonato, 2018; Procházka et al., 2018; Scharff et al., 2003). However, it is still common practice to carry out these analyses on the basis of the number of observed species. Estimations and direct observations have rarely been compared to assess the extent of under‐representation and whether this is evenly distributed among different subsamples. Especially in applied conservational practice, there are benefits to working with observed data, mainly because calculating the estimates is time‐consuming and requires a higher expertise in statistics. To assess to what extent it may be reasonable to work with observed species counts and to what extent published studies based on them can be regarded as reliable sources, we assessed whether different estimators of species richness and the observed number of species of Coleoptera in the canopy of the Leipzig floodplain forest provided the same pattern of species richness and shared species across subsets of data. The subsets of data were the three main tree species at two different strata of the canopy. Specifically, we addressed the following questions: (a) How strongly do observed and estimated species richness differ? (b) Are the differences similar among tree species, canopy strata, or seasons? and (c) How do observed and estimated numbers of shared species among communities differ?

## 2 MATERIALS AND METHODS

### 2.1 Sampling site and scheme

Saxony is a state in the east of Germany with borders with Poland and the Czech Republic. The climate is classified as warm temperate climate with year‐round humidity (Kottek et al., 2006). The main river in western Saxony is the Weisse Elster, which branches off into the Luppe and is joined by the Pleiße in the area of Leipzig. This river system creates the Leipzig floodplain forest. Sampling was carried out by means of the Leipzig Canopy Crane. It is a revolving tower crane (Liebherr 71 EC), which is 40 m high, has a beam length of 40 m, and spans an area of 16,500 m^2^. With this nondestructive access to the canopy we are able to minimize negative effects of destructive methods, such as fogging or logging. The forest covered by the crane is a near‐natural deciduous forest, which largely consists of *Quercus robur* L., *Fraxinus excelsior* L., *Tilia cordata* L., *Acer pseudoplatanus* L., *Ulmus laevis* L., and *Carpinus betulus* L. We focused on the dominant tree species *Q. robur*, *T. cordata,* and *F. excelsior* and installed a total of 24 omnidirectional window flight intercept traps (Wilkening et al., 1981) in two strata (20 and 25 m height). Of *T. cordata* and *F. excelsior,* four trees were sampled, each with one trap in the lower and one in the upper canopy. Of *Q. robur*, and only three individual trees were accessible. To keep the number of traps per species comparable, we installed two flight interception traps per stratum on the largest oak, one each on opposing sides of the canopy. The traps consisted of an upper sampling unit, a lower sampling unit, and two inter‐crossed plexiglass panels. The upper and lower sampling unit each consisted of a sampling container filled with diethylene glycol. Sampling was performed biweekly from 31 March 2016 to 28 September 2016. In the following, we refer to these sampling dates as trap collections. The sampling period was expected to be the main activity period for arthropods in our study area. It is common practice to collect the traps monthly (e.g., Bouget et al., 2008; Knuff et al., 2019), but our biweekly collection interval was chosen to minimize evaporation of sampling liquids. The lower sampling units of the two traps of one tree of *F. excelsior* in both strata were lost due to a storm between 21 July 2016 and 04 August 2016, but were replaced after the storm and in use throughout the rest of the season.

### 2.2 Species determination and composition

All beetles were determined to the species level using mainly Freude, Harde, Lohse (1964–1983), and supplements. For 37 taxa, neither determination to the species level nor a meaningful classification to morphospecies was possible (mainly different species of Aleocharinae). These taxa were excluded from further analyses.

### 2.3 Observed and estimated species richness across the studied strata and tree species

The R‐package iNEXT was used for species richness estimations (Hsieh et al., 2016). We compared the asymptotic estimator Chao‐1 to the nonasymptotic estimate from rarefaction and extrapolation sampling curves for Hill number *q* = 0. We extrapolated up to 30 trap collections, which is approximately twice of our actual number of trap collections. We always used the value for 30 trap collections, as at this point the slope of the curve was the least steep.

We calculated all estimates separately for either strata or tree species. For each subset, we used the biweekly dates of the trap collections as replications in time. The single traps within a given tree species or stratum and trap collection were pooled.

When $N_{\mathrm{obs}}$ is the number of species observed and $N_{\mathrm{est}}$ is the number of species estimated, sample completeness was calculated as $N_{\mathrm{obs}}/N_{\mathrm{est}}$ (note, terminology follows Chao et al. (2020), various earlier publications called this term sample coverage). We calculated sample completeness separately for all subsets and selected estimators. To assess whether the influence of the subset on the sample completeness was significant, we carried out two‐way ANOVAs, separately for tree species and strata.

### 2.4 Ecological inferences of differences in species richness per stratum and tree species

To assess whether the effect of stratification and tree species on species richness differed depending on the used model, we carried out two‐way ANOVAs, one with estimator and stratum and one with estimator and tree species as the categorical independent variables. The observed values were included as one model, and a Tukey post hoc test was run to determine whether the observed values differed significantly from the estimated values. Interactions between the variables could not be calculated, as there was not enough data and thus the degrees of freedom were not sufficient to determine the residual variability or form standard errors. For interaction models, more trap collections per season would be needed. The analyses were carried out in base R (R Core Team, 2019).

### 2.5 Observed and estimated number of shared species across studied strata and tree species

To estimate the number of species shared between upper canopy and lower canopy, we used the approach developed by Chao et al. (2000) as implemented in the R‐package SpadeR (Chao et al., 2016a). To estimate the number of species that are shared between pairs of tree species only and all three tree species, we developed a new approach. We first note that the number of species shared between *Q. robur* and *T. cordata* (Q∩T) consists of the number of species shared only between the two species (QT) and the number of species shared among all three species (QFT). Thus:

(1) Q∩T=QT+QFT.

Similarly, for *Q. robur* and *F. excelsior*:

(2) Q∩F=QF+QFT,

and for *F. excelsior* and *T. cordata*:

(3) F∩T=FT+QFT.

By combining the datasets for *T. cordata* and *F. excelsior*, we estimated the number of species shared by this combined dataset with *Q. robur* ((F∪T)∩Q), which consists of the species that *Q. robur* shares only with *F. excelsior* (QF), the species that *Q. robur* shares only with *T. cordata* (QT), and the species shared by all three species (QFT). Thus:

(4) $$\left(F\cup T\right)\cap Q=QF+QT+QFT.$$

The left side of Equations (1)–(4) can be estimated using the method of Chao et al. (2000) from the R‐Package SpadeR (Chao et al., 2016a) to estimate the number of shared species between two entities. By solving Equations (1) and (2) for QT and QF, respectively, inserting them in Equation (4) and solving the resulting equation for QFT, we will obtain the number of species shared by all three species as:

(5) $$QFT=Q\cap F+Q\cap T‐\left(F\cup T\right)\cap Q.$$

Inserting the results for Equation (5) into Equations ((1), (2), (3))–((1), (2), (3)), we will obtain the number of species shared between two tree species but not with the third tree species:

QT=Q∩T‐QFT,

QF=Q∩F‐QFT,and

FT=F∩T‐QFT.

Finally, we can obtain the number of species that are present only on one tree species (*Fo*, *Qo*, *To*) by subtracting the number of species that the tree shares with other tree species from the estimated number of species for the focal tree species (*F*, *Q*, *T*):

Fo=F‐QF‐FT‐QFT,Qo=Q‐QF‐QT‐QFT,andTo=T‐QT‐FT‐QFT.

Species observed and estimated to be shared between the different strata and tree species were compared and visualized by Venn diagrams using the R‐package “VennDiagram” (Chen, 2018).

To test whether the number of beetle species falling into the categories shared between none (one category for each tree species), two (three categories), and three tree species (one category) differed significantly between the observed and estimated values, we summed the estimates for all categories and calculated the percentages that fell into each category. By multiplying the total number of observed species with these percentages, we calculated the expected number of observed species for each category and compared the observed with the expected numbers with a chi‐square test. We rounded all estimates to the nearest integer. The tested null hypothesis was that the distribution of observed values across categories followed the distribution of expected values derived from the estimated values.

## 3 RESULTS

### 3.1 Species determination and composition

In 2016, a total of 6,021 individuals of the order Coleoptera were collected. Five hundred and fifty‐three individuals could not be determined to the species level and were excluded from subsequent analyses. The remaining 5,187 individuals belonged to 279 species of 51 families.

### 3.2 Observed and estimated species richness across the studied strata and tree species

**FIGURE 1:**
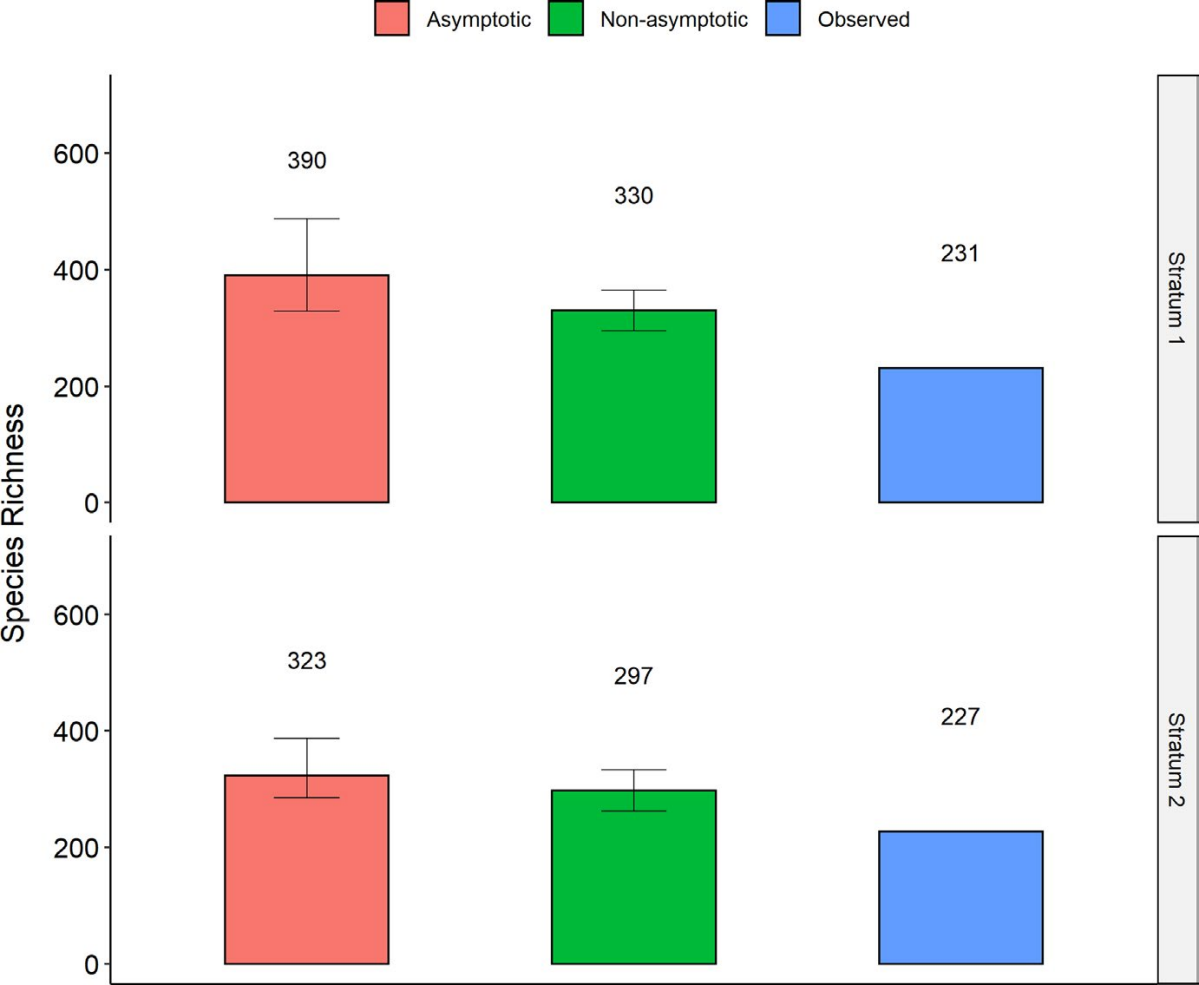
Number of observed species and estimated species richness using asymptotic and nonasymptotic estimators for the different canopy strata. Stratum 1 refers to the upper canopy, and stratum 2 refers to the lower canopy. Confidence intervals are indicated as error bars

**FIGURE 2:**
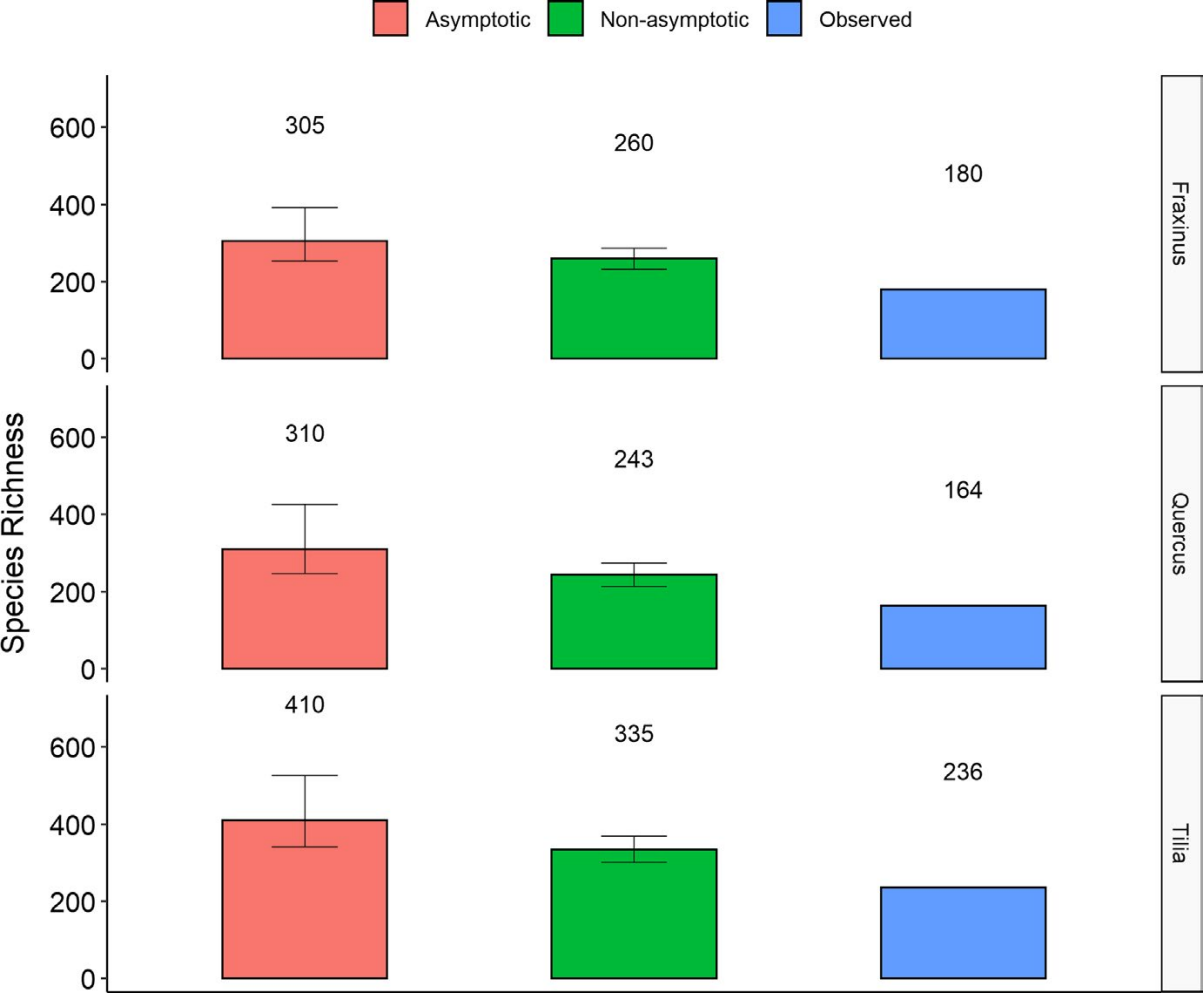
Number of observed species and estimated species richness using asymptotic and nonasymptotic estimators for the different tree species. Confidence intervals are indicated as error bars

The confidence intervals of estimated species richness overlapped considerably among each other for all subsets of data (Figures 1 and 2).

In terms of species richness, the ranking patterns obtained for host tree species and strata were mostly the same for the estimators and for the observed number of species. Only the asymptotic estimator returned a higher value for *Q. robur* than for *F. excelsior*, while the observed values and the nonasymptotic estimator showed the opposite pattern (but the difference between the two species was small for both estimators) (Figure 2).

The observed and the estimated values of species richness were higher in the upper canopy than in the lower canopy. Confidence intervals were, however, overlapping (Figure 1).

For both estimators and the observed value, species richness was higher in *T. cordata* than in the other tree species. The observed values and the nonasymptotic estimators showed the lowest richness in *Q. robur,* while the asymptotic estimator showed the lowest richness in *F. excelsior* (Figure 2).

The sample completeness of both estimators was between 0.5 and 0.8 for all subsets. It was always higher for the nonasymptotic estimators (Table 1).

**TABLE 1:** Sample completeness of all subsets for all estimators

Factor	Subset	Observed	Estimator type	Estimator	Sample completeness
Stratum	Lower canopy	227.0	Asymptotic	323.1	0.7
Stratum	Lower canopy	227.0	Nonasymptotic	297.5	0.8
Stratum	Upper canopy	231.0	Asymptotic	389.7	0.6
Stratum	Upper canopy	231.0	Nonasymptotic	330.4	0.7
Tree species	*Fraxinus*	180.0	Asymptotic	304.7	0.6
Tree species	*Fraxinus*	180.0	Nonasymptotic	259.6	0.7
Tree species	*Quercus*	164.0	Asymptotic	310.2	0.5
Tree species	*Quercus*	164.0	Nonasymptotic	243.1	0.7
Tree species	*Tilia*	236.0	Asymptotic	410.4	0.6
Tree species	*Tilia*	236.0	Nonasymptotic	335.2	0.7

Sample completeness did not differ among strata or among tree species. However, for the tree species the sample completeness differed significantly between the estimators (Table 2).

**TABLE 2:** Results of two‐way ANOVA for the influence of the used estimator and subset of data on sample completeness. The tested subsets are the sampled canopy strata and tree species, respectively

Subsets	Parameters	*df*	Sums of squares	Mean squares	*F* statistics	*p*‐Values
Stratum	Estimator type	1	0.01	0.01	13.24	.17
	Subset	1	0.01	0.01	14.36	.16
	Residuals	1	0.00	0.00		
Tree species	Estimator type	1	0.02	0.02	98.99	.01
	Subset	2	0.00	0.00	4.25	.19
	Residuals	2	0.00	0.00		

### 3.3 Ecological inferences of differences in species richness per stratum and tree species

The effect of stratum was insignificant, but the effect of the used estimator (the observed values are included in the parameter “estimator”) on species richness was marginally significant (Table 3).

**TABLE 3:** Results of two‐way ANOVA for the influence of stratum and estimator on estimated species richness

Parameters	*df*	Sums of squares	Mean squares	*F* statistics	*p*‐Values
Stratum	1	1,802.67	1,802.67	3.63	.20
Estimator	2	16,830.33	8,415.17	16.93	.06
Residuals	2	994.33	497.17		

When taking into account the strata, the differences between the nonasymptotic and asymptotic estimators as well as the differences between the observed values and the nonasymptotic estimator were insignificant (Tukey's HSD *p*‐value .329 and .113, respectively), while the differences between the observed values and the asymptotic estimator were slightly significant (Tukey's HSD *p*‐value .053).

The ANOVA showed a highly significant effect of tree species and the used estimator (the observed values are here included in the parameter “estimator”) on species richness (Table 4). Significant differences in species richness among tree species were found between *T. cordata* and the other two tree species (*Q. robur* and *F. excelsior*) (Tukey's HSD *p*‐value .002 and .004, respectively) but not between *Q. robur* and *F. excelsior* (Tukey's HSD *p*‐value .67).

**TABLE 4:** Results of two‐way ANOVA for the influence of tree species and estimator on estimated species richness

Parameters	*df*	Sums of squares	Mean squares	*F* statistics	*p*‐Values
Tree species	2	14,019.56	7,009.78	43.24	.00
Estimator	2	33,284.22	16,642.11	102.66	.00
Residuals	4	648.44	162.11		

The differences between the nonasymptotic and asymptotic estimators were highly significant (Tukey's HSD *p*‐value .009) as were the differences between the observed and estimated values (Tukey's HSD *p*‐values .003 and 0 for the comparison of the observed values with the nonasymptotic and the asymptotic estimator, respectively).

### 3.4 Observed and estimated number of shared species across studied strata and tree species

**FIGURE 3:**
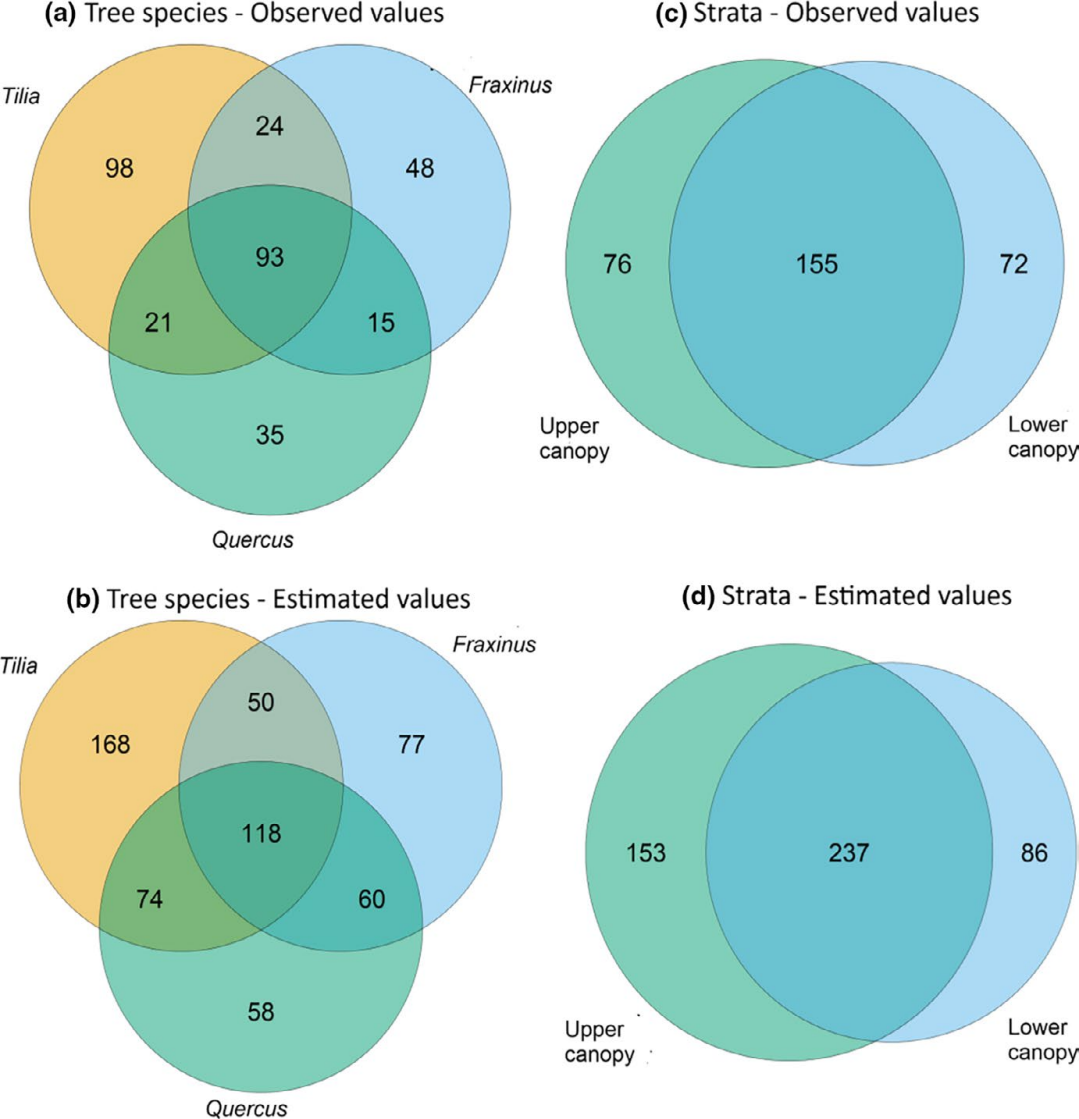
Venn diagram showing (a) the observed number of species shared between the three main tree species (*F. excelsior*, *Q. robur*, *T. cordata*), (b) the estimated number of species shared between the three main tree species (*F. excelsior*, *Q. robur*, *T. cordata*), (c) the observed number of species shared between the two canopy strata, and (d) the estimated number of species shared between the two canopy strata

Using Venn diagrams, we compared the observed and estimated numbers of shared beetle species between the tree species (*Q. robur*, *F. excelsior*, *T. cordata*) and the canopy strata (Figure 3). The estimated and observed results showed that more species were unique to *T. cordata* than to any other category. The number of observed species shared between *T. cordata* and any of the other tree species was similar and higher than the number of observed species shared between *Q. robur* and *F. excelsior*. The estimated data showed a different result. Here, the number of species shared between *Q. robur* and any of the other tree species was highest (Figure 3b). The number of unique beetle species was highest for *T. cordata* and lowest in *Q. robur* for the observed and estimated values (Figure 3a,b).

More beetle species were shared between the strata than were unique to any stratum in both the observed numbers (Figure 3c) and estimated numbers (Figure 3d). The number of species unique for each stratum was similar in the observed values (Figure 3c), but in the estimated values the upper canopy showed a number of unique species almost twice as high as the lower canopy (Figure 3d).

The number of species expected for each category under the null hypotheses differed significantly from the observed values ($\chi_{5}^{2}$ = 33.861, *p* = 0). We therefore rejected the null hypothesis and concluded that the observed distribution differed significantly from the expected distribution (Table 5).

**TABLE 5:** Estimated, observed, and expected species unique to and shared among tree species. Proportion estimated shows the proportion of the summed number of species across all communities, which is covered by the given subset. Expected shows the number of species that would have been expected to be observed in a given subset based on that proportion

Communities	Estimated	Proportion estimated	Observed	Expected
*Quercus* only	58	0.10	35	32
*Fraxinus* only	77	0.13	48	42
*Tilia* only	168	0.28	98	93
*Quercus Tilia*	74	0.12	21	41
*Quercus Fraxinus*	60	0.10	15	33
*Fraxinus Tilia*	50	0.08	24	28
All	118	0.19	93	65

## 4 DISCUSSION

We found that while using the observed number of species as a measure of species richness would significantly underestimate the species richness of Coleoptera in the Leipzig floodplain forest, the observed data could be used for comparative analyses of tree species or strata, as the sample completeness was similar among tree species and strata.

The missed species were most probably rare species and transient species, which are likely to be missed due to their low detection probability (Chao et al., 2016b). This possibility of missing species when working with observed data demonstrates the importance of species richness estimation models when working with field study datasets. This finding is in line with other studies, which have shown that in forest ecosystems with a high biodiversity the observed richness is severely under‐representing alpha diversity (Oliveira et al., 2016; Peretti & Bonato, 2018; Scharff et al., 2003). The significant difference between our estimated and observed number of species could be explained by three effects: methodological, spatial, and temporal edge effect. When working with arthropods, the methodological effect is especially important to consider. This is because detection probability in arthropods varies greatly, without necessarily being related to the species' abundance (Longino et al., 2002). Arthropods also exhibit a particularly high phenological heterogeneity due to their short life cycles. This difficulty in defining a homogeneous species pool in the field also limits the accuracy of estimators (Follner & Henle, 2001).

The observed data represented all studied subsamples equally reliably. If the tree species or strata would show significantly different sample completeness, the ranking would differ between the estimated and observed values for species richness. As this was not the case, observed data could be used for ranking comparisons of strata or tree species in our study system. As long as the study system and sampling design are similar to ours, this indicates that relative comparisons with earlier studies, in which no estimators have been used, are likely reliable.

Neither vertical stratification nor sampled tree species had a significant influence on beetle species richness. This finding is in line with similar studies on beetle species richness in temperate forests (Müller & Goßner, 2010; Procházka et al., 2018). It is a further hint that vertical stratification might not be as strongly shaping the alpha diversity of communities in temperate forests as in the tropics. We need to consider that the compared strata are both within the canopy which limits the impact of stratification.

Observed values could be used for qualitative comparative ranking of shared beetle species between the main tree species in the floodplain forest, as the ranking patterns of shared beetle species between the tree species were largely similar. Notwithstanding, the observed values differed significantly from the expected distribution of shared species. For in‐depth analyses of species composition in our study system, estimated values should be used. The pattern of species shared between the two canopy strata differed crucially between the observed and the estimated values, which means that the observed values cannot be used for analyses of community composition of the canopy strata and estimated values should always be used.

*T. cordata* showed a species richness higher than the other studied tree species, which is likely caused by the fact that the flowers of *T. cordata* are highly attractive to a number of insects, including beetles (Anderson, 1976). *Q. robur*, a tree that was historically one of the dominant tree species of the Leipzig floodplain forest (Klimo & Hager, 2001), did not show higher species richness than *F. excelsior* or *T. cordata*, two tree species which became common in the floodplain forest only more recently (Klimo & Hager, 2001). This implies that to protect the beetle communities, it is not meaningful to concentrate on single tree species. It is important to note that the studied tree species are typical floodplain forest species, and this statement cannot be extended to introduced species such as *Acer pseudoplatanus* or *Quercus rubra*. Earlier studies suggest that oaks harbor a richer arthropod biodiversity than other tree species (Schmidl, 2006; Unterseher et al., 2007). As the present study is based on a single year, further sampling would be needed to determine whether this greater species richness on oak in earlier studies reflects annual variability or was an artifact, possibly due to the use of observed values that do not account for detectability. For all observations, we need to consider that our result might be partly caused by the fact that flight interception traps capture individuals while they are on the move, not while they are feeding, so microhabitat specificity is lower (Bouget et al., 2008).

Even though the use of flight interception traps could bias the number of unique species due to their dependence on moving individuals, we believe that there is good evidence that they can still be used for true ecological distribution patterns of specialized beetles. For example, *Protaetia speciosissima* was among the species restricted to the upper canopy in our samples. The literature proves that it is in fact a canopy specialist, which is known to fly mostly high above the forest canopy (Lillig, 2012). The larvae of this species develop mostly in rotten branches and tree hollows several meters above ground (Rößner, 2012). Likewise, we found some interesting specialists among the beetle species which were unique to one of the tree species. *Opilo pallidus*, in general a quiet rare species which was the most common species among the ones only found in *Q. robur*, is linked to small branches of old oaks, where its larvae hunt other insect larvae. It is furthermore also a canopy specialist and was coherently not found in the understory (Harde et al., 1979). *Ernoporus tiliae*, the species that was the most common one among the species only found in *T. cordata*, is found only when stands of *Tilia* spp. are present and breeds in fresh dead wood of limes (Harde et al., 1981). These examples of these specialists illustrate that further research on the beta diversity among tree species sampled is highly relevant for the ecological characterization of the floodplain forests.

## 5 CONCLUSION

For qualitative comparative analyses of species richness, the observed values could be used in most cases, allowing a cautious comparison with published analyses that were based on observed values. For quantitative analyses, however, the use of species richness estimators is essential, and contrary to current practice, observed data should not be used.

## CONFLICT OF INTEREST

We know of no conflicts of interest associated with this publication.

## AUTHOR CONTRIBUTIONS

**Nora Haack:** Conceptualization (equal); Data curation (lead); Formal analysis (equal); Investigation (equal); Methodology (equal); Project administration (equal); Software (equal); Visualization (lead); Writing‐original draft (lead); Writing‐review & editing (lead). **Annegret Grimm‐Seyfarth:** Conceptualization (equal); Methodology (equal); Software (equal); Writing‐original draft (supporting); Writing‐review & editing (supporting). **Martin Schlegel:** Conceptualization (supporting); Funding acquisition (equal); Project administration (equal); Resources (lead); Supervision (equal); Writing‐original draft (supporting); Writing‐review & editing (supporting). **Christian Wirth:** Funding acquisition (equal); Project administration (supporting); Resources (supporting); Supervision (supporting); Writing‐original draft (supporting); Writing‐review & editing (supporting). **Detlef Bernhard:** Conceptualization (supporting); Data curation (supporting); Funding acquisition (supporting); Supervision (supporting); Writing‐original draft (supporting); Writing‐review & editing (supporting). **Ingo Brunk:** Data curation (equal); Writing‐review & editing (supporting). **Klaus Henle:** Conceptualization (equal); Formal analysis (equal); Investigation (equal); Methodology (equal); Supervision (equal); Writing‐original draft (supporting); Writing‐review & editing (supporting).

## Figures and Tables

**FIGURE A1:**
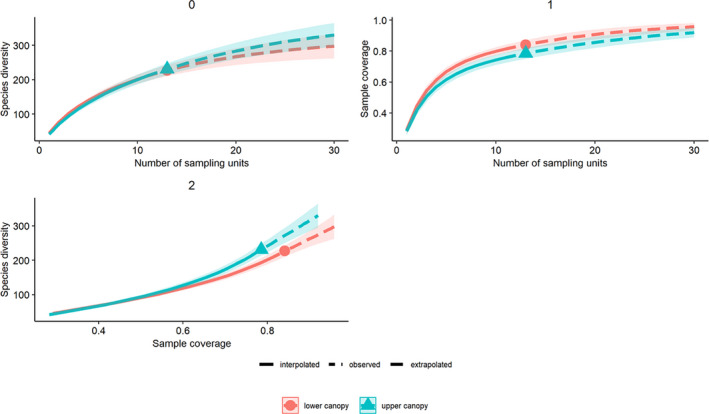
Sample‐size‐based and coverage‐based rarefaction (solid line segment) and extrapolation (dotted line segments) sampling curves with 95% confidence intervals (shaded areas) for the sampled strata. The solid dots/triangles represent the reference samples

**FIGURE A2:**
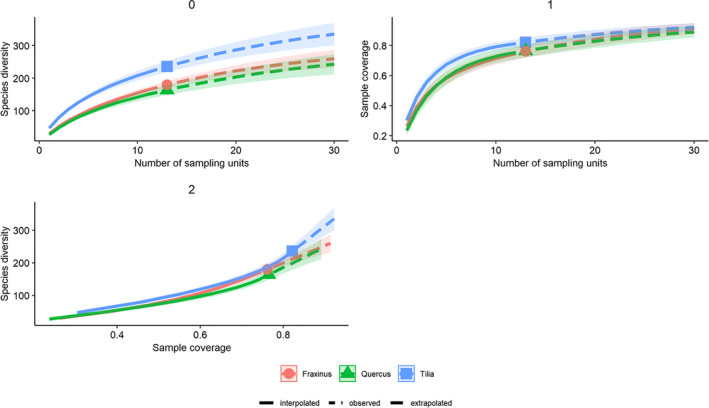
Sample‐size‐based and coverage‐based rarefaction (solid line segment) and extrapolation (dotted line segments) sampling curves with 95% confidence intervals (shaded areas) for the sampled tree species. The solid dots/triangles represent the reference samples

## Data Availability

Our dataset is stored in the Dryad data portal, where it will be accessible for the wider public. The DOI is https://doi.org/10.5061/dryad.d7wm37q0g.
